# Beyond seeds: Revealing the clonal reproduction of *Bulbostylis paradoxa* as a persistence mechanism in tropical savannas

**DOI:** 10.1002/ecy.70225

**Published:** 2025-10-08

**Authors:** Hudson G. V. Fontenele, Ana L. D. Lacerda, Heloisa S. Miranda

**Affiliations:** ^1^ Departamento de Ecologia Instituto de Ciências Biológicas, Universidade de Brasília Brasília Brazil

**Keywords:** Cerrado, clonality, ground layer, open ecosystems, tropical grasslands, vegetative reproduction

There is significant unawareness regarding the ecological strategies (e.g., post‐fire flowering, clonal reproduction, fire‐stimulated recruitment) of the ground layer in tropical grassy biomes. Despite its critical role in maintaining ecosystem stability (Bond, 2021; Pausas & Bond, 2020), the non‐woody component remains understudied, with far greater attention directed toward woody species than toward the life history of graminoids, forbs, and subshrubs (Buisson et al., 2021). Indeed, these species pose a considerable challenge to science, as observing and characterizing their biology often require years of rigorous fieldwork, given their long‐lived habits and the old‐growth assemblages that define these communities (Veldman et al., 2015). Consequently, elucidating the mechanisms that drive population dynamics and shape communities remains one of the most pressing challenges in grassland and savanna research, potentially hindering effective management and conservation efforts (Buisson et al., 2021). Similar challenges are evident in the Cerrado ecoregion (Brazilian mesic open ecosystems), where the ground layer harbors approximately 60% of the region's native species (JBRJ, 2025) but has only recently emerged as a focus for both basic and applied research (Durigan et al., 2020; Pilon et al., 2021), with some of its life strategies being uncovered only in the last few years (Maracahipes et al., 2024).

Surely, among the most remarkable strategies observed in the Cerrado is that of *Bulbostylis paradoxa* (Spreng.) Lindm., an iconic sedge (Cyperaceae) that has gained recognition for blooming within 24 h after any fire (Fidelis et al., 2019). This extraordinary strategy has established *B. paradoxa* as a flagship species in discussions about fire ecology in the Cerrado, sparking considerable scientific interest that has led to investigations into the species' morphology, ecophysiology, and reproductive biology. Early studies suggested that the species' flowering was fire‐dependent (Fidelis et al., 2019) and raised questions about nutrient reserves, anatomic adaptations, and the triggers that supported its speedy blooming. Detailed investigations revealed fast‐mobilizing carbohydrates (Rosalem et al., 2022) and protective leaf sheaths (Rosalem et al., 2025) as adaptations enabling flowering even after complete charring during the dry season. Then, further research clarified that flowering is actually fire‐stimulated rather than fire‐dependent, with rainfall also serving as a trigger during fire‐free periods (Miranda et al., 2024). However, still, none of these works have explored one of the most prevalent strategies within the tropical grassy biomes: the ability to reproduce clonally (Veldman et al., 2015). While older studies have confirmed this species' ability to spread vegetatively (Rodrigues & Estelita, 2009; Weber, 1963), the detailed understanding of its asexual reproduction and its subsequent ecological relevance has been overshadowed by the astonishing post‐fire flowering. Therefore, taking part in the effort to advance our understanding of this flagship species, we contribute with observations revealing its clonal reproduction mechanism.

*Bulbostylis paradoxa* (Figure 1) is easily distinguished from typical sedges (descriptions from Weber, 1963). Its individuals develop a central, aboveground caudex that grows vertically from the soil (parallel to the longitudinal axis), with axillary buds at the apex differentiating to produce leaves and inflorescences. The caudex is encased in a dense external mantle formed by persistent remnant leaf sheaths from previous growing seasons, which protects the internal meristematic tissues from fire (Rosalem et al., 2025) and also absorbs rainfall, potentially storing double its weight when water‐saturated (Weber, 1963). Primary rooting is shallow, with several thin roots emerging from the base of the caudex to anchor the plant to the soil (rhizomes are absent). Additionally, a secondary rooting system develops within the caudex, forming an inner diffuse adventitious root system just beneath the mantle border, which collects the moisture retained by the mantle (Mora‐Osejo, 1989; Porembski, 2006; Weber, 1963). Often, axillary buds elongate and thicken to form lateral sympodial units, resulting in individuals with multiple “branches” that remain interconnected through the main caudex. These two features—the inner root system and the sympodial units—underpin the species' clonal potential (Porembski, 2006).

**FIGURE 1:**
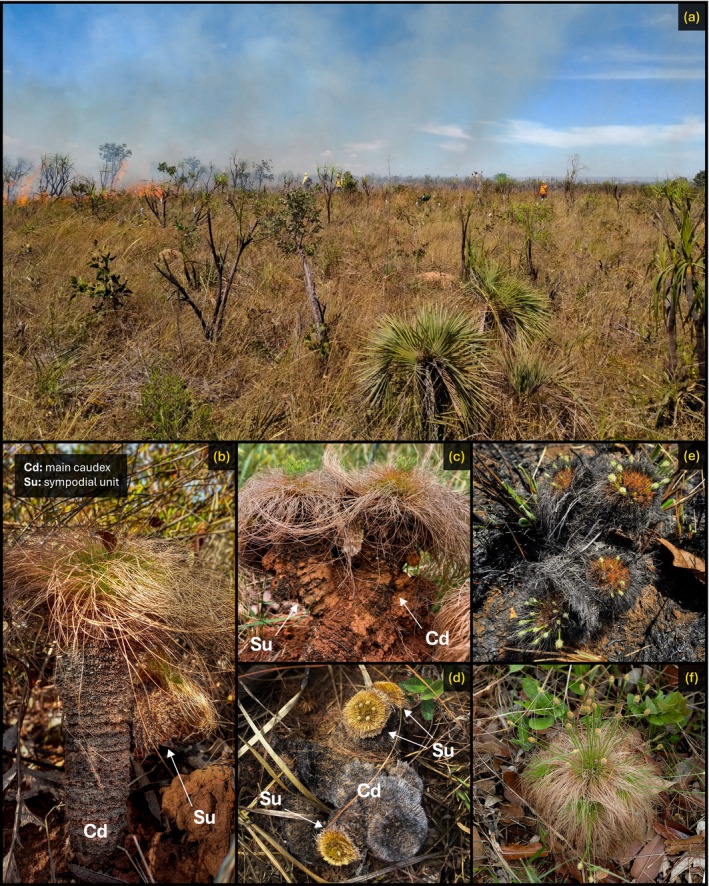
Photographs of the sedge *Bulbostylis paradoxa* (Spreng.) Lindm., detailing (a) an old‐growth Cerrado grassland where it occurs; (b–d) general morphology comprised of a caudex (Cd) and sympodial units (Su); and (e, f) flowering with and without fire. Photo credits: Hudson G. V. Fontenele (a), Ana L. D. Lacerda (b, c, f), Heloisa S. Miranda (d, f).

Over the past 7 years, we have monitored two distinct populations (separated by 10 km) occurring in two old‐growth grasslands located in Brasília, Central Brazil (Área Alfa da Marinha do Brasil, 16°00′57″ S–47°55′43″ W; Reserva Ecológica do IBGE, 15°57′10″ S–47°52′10″ W; a detailed site description may be found in Appendix S1). As we tagged our monitored individuals, we frequently observed multiple tussocks growing in close proximity, often forming dense clusters or trails (Figure 2). This aggregated distribution suggested the potential for clonal reproduction (Klimešová et al., 2021). We noted that some individuals differed from the species' typical morphology, with caudexes developing parallel to the soil rather than vertically. These horizontal individuals did not grow along their longitudinal axis but instead produced leaves and roots perpendicularly (vertically) while the caudex remained horizontally oriented (Figure 2). The longitudinal ends of these horizontal individuals exhibited visible damage scars, indicating fragile sections that had previously been attached to a larger structure. Additionally, some of these plants could be easily lifted from the soil, suggesting shallow and recent rooting. By comparing these horizontal individuals with the standard vertical ones, we identified the presence of sympodial units, which varied in size and angle relative to the vertical axis (Figure 1), with some reaching lengths of up to 20 cm. Some vertical individuals also displayed damage scars, indicating the loss of sympodial units that had broken off and fallen nearby. These observations led us to hypothesize a fragmentation mechanism (Klimešová et al., 2021; Porembski, 2006) through which clones are produced by the detachment of parts from the parent plant.

**FIGURE 2:**
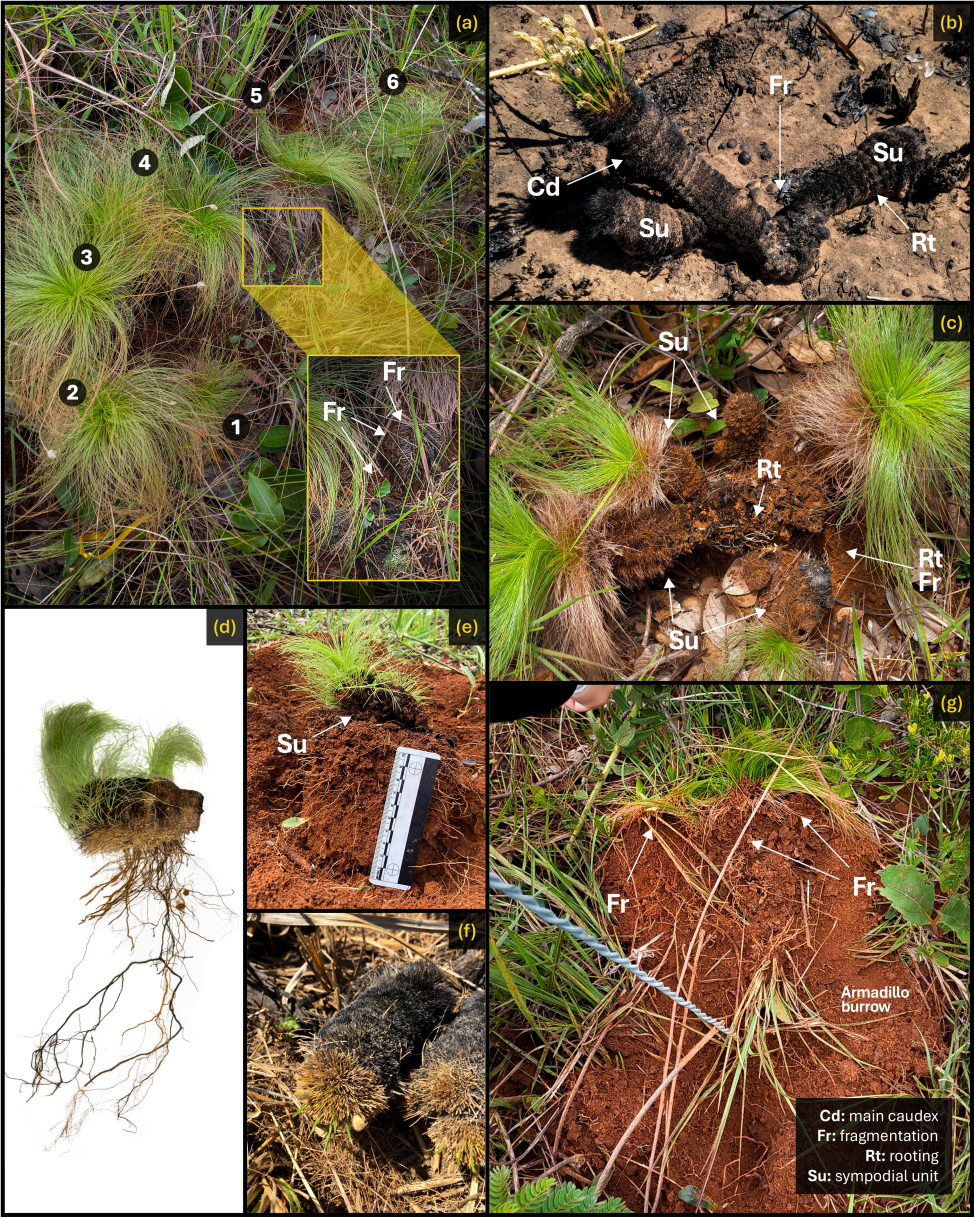
Photographs showing the clonal reproduction through sympodial fragmentation of *Bulbostylis paradoxa* (Spreng.) Lindm: (a) trail of individuals (numbered) displaying signs of past connections, including a recent fragmentation (Fr; zoomed in) showing rooting (Rt); (b, c) individuals with multiple sympodial units (Su) and fragmentation events, with units rooted nearby; (d, e) old‐established clone with a horizontal caudex (Cd) and leaves growing perpendicularly rather than longitudinally; (f) newly established clone with leaves still growing from the end of the caudex; (g) armadillo foraging, which creates new viable propagules. Photo credits: Ana L. D. Lacerda (a, c, g), Hudson G. V. Fontenele (b), Heloisa S. Miranda (d, e, f).

We have termed this mechanism “sympodial fragmentation,” which appears to occur in three phases (Figure 3). During **phase I**, individuals develop sympodial units that grow at an incline relative to the main vertical axis. Multiple units may form before fragmentation occurs, and they function similarly to the main caudex, producing leaves, flowers, and an inner root system. The growth of these lateral units can span several decades until they reach sufficient length to detach from the main caudex. Among our monitored individuals (*n* = 70 in each site), 41% and 49% had at least one lateral head, suggesting that nearly half of the populations have the potential to reproduce clonally.

**FIGURE 3:**
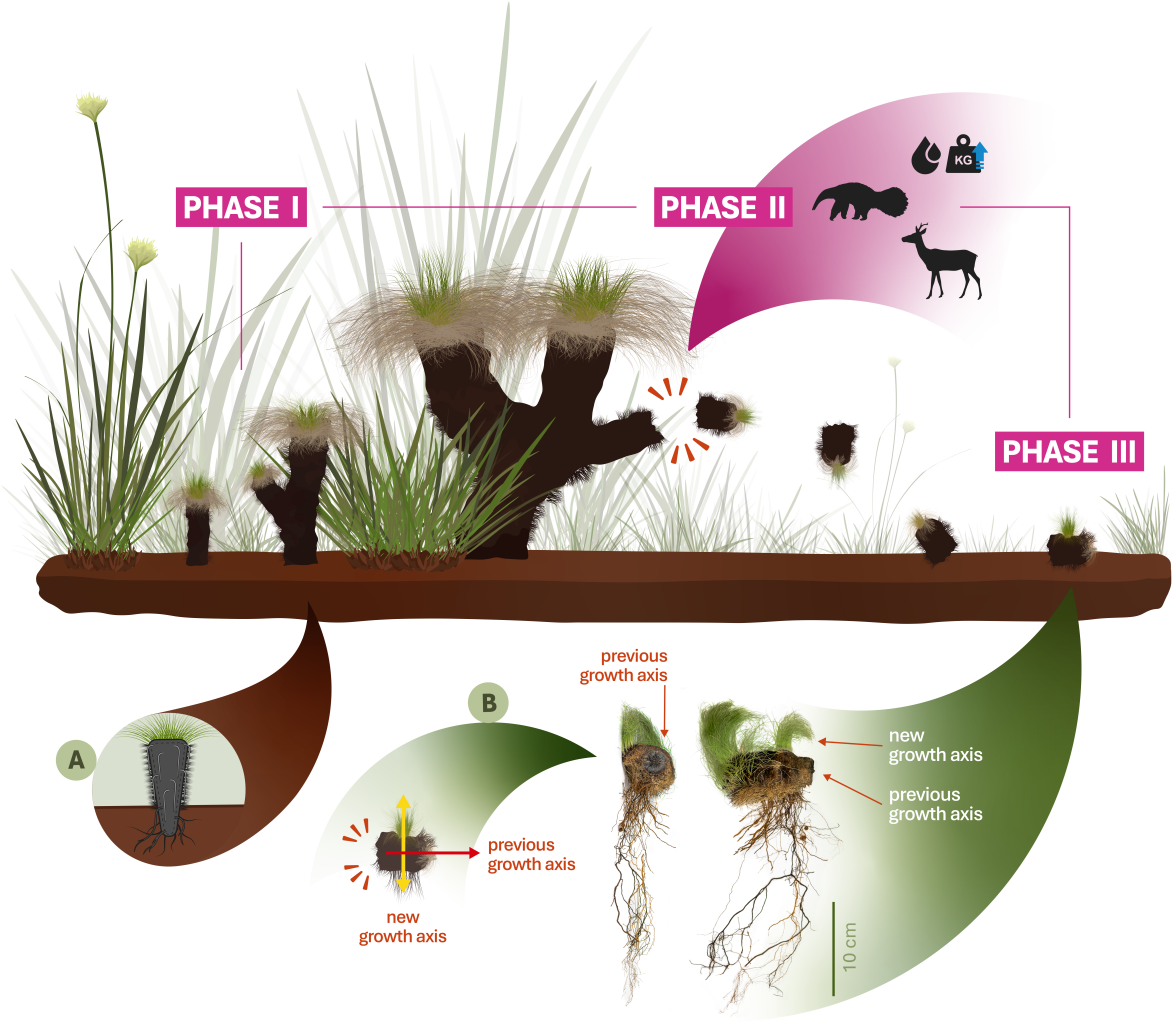
Illustrations of the stages of the clonal reproduction mechanism of *Bulbostylis paradoxa* (Spreng.) Lindm. Individuals develop functional sympodial units (phase I), which detach from the main caudex due to external forces (phase II) and land on suitable, open soil, rooting themselves to form a new individual that grows parallel to the soil (phase III and B). This mechanism is supported by the species' inner root system and water‐absorbing mantle, shown in A. Illustration credit: Hudson G. V. Fontenele; water droplet, weight icons designed by Freepik from Flaticon (flaticon.com). Photo credit: Heloisa S. Miranda.

In **phase II**, one of the lateral units detaches from the main caudex, creating a mobile propagule capable of establishing a physiologically independent individual elsewhere. However, individuals cannot detach units at will, and it probably results from external mechanical forces. The delicate structural integrity of the caudex makes it highly susceptible to crushing under strong external forces (Porembski, 2006; Weber, 1963), and we propose three factors causing detachment: (1) Grazing by herbivores, such as deer, could easily detach sympodial units. This would be most relevant in the immediate post‐fire environment when *B. paradoxa*'s rapid blooming and leaf flushing provide a critical food source. However, as the leaves mature and become tougher, they become less attractive to herbivores, reducing the likelihood of this factor after the initial post‐fire weeks. (2) Mechanical contact with medium‐ to large‐sized animals, such as anteaters, armadillos, rheas, and tapirs, could potentially fragment individuals and displace sympodial units over greater distances. This phenomenon is the most likely and has been observed with armadillos, which fragment individuals while foraging through the soil during burrowing. (3) Water absorption by the mantle, which can double a unit's weight, could alter tissue turgor and potentially lead to fragmentation if internal structures become sufficiently fragilized. However, this is likely a rare phenomenon, reserved for sympodial units with thin bases or already compromised stems, as the increased weight alone should not break thick‐stemmed caudexes. Nevertheless, this process could also facilitate other fragmentation mechanisms by making the structures more prone to breakage during mechanical contact.

In **phase III**, the fragment establishes horizontally, producing leaves and roots. The position of leaf flushing can indicate the relative age of a clone's establishment, as leaves emerging parallel to the ground likely reflect remnants of the original growth axis, while those emerging on the side of the fallen caudex and growing perpendicular to the ground suggest an older clone (Figure 3). Importantly, not all detachments result in successful establishment. A few weeks after armadillos fragmented a couple of our monitored individuals, we found the detached units above the grasses, completely dry, with loose leaves on the ground and no evidence of rooting, suggesting the death of the individuals. Therefore, for successful establishment, the fragment must fall into an open vegetation gap during periods of sustained rainfall, allowing the inner root system to reach the soil and establish itself.

Clonality is a frequent trait in seasonal, nutrient‐poor, fire‐prone ecosystems such as tropical grasslands and savannas (Veldman et al., 2015). Yet, the exact mechanisms of multiplication often remain unclear. *Bulbostylis paradoxa* was reported to reproduce clonally (Rodrigues & Estelita, 2009), but its mechanism remained uncertain because, unlike typical graminoids, it does not form structures for rhizomatous (rhizomes) or stoloniferous (tillers) spread. Instead, it exploits its unique architecture to create mobile propagules that can establish further than rhizomes and with greater security than stolons or seedlings. Initially, the fragmented branch may fall and roll, increasing the horizontal spread of the population. Then, the detached sympodial unit, pre‐equipped with functional inner roots, carbohydrate reserves, and a water‐absorbing mantle (Rodrigues & Estelita, 2009; Rosalem et al., 2022; Weber, 1963), bypasses the vulnerable early stages that constrain recruitment by seeds in the harsh savanna environments (Porembski, 2006), ensuring higher establishment success. Later, the pre‐formed caudex provides fire protection even to recently detached clones (Rosalem et al., 2025; Weber, 1963), giving these clonal fragments a critical advantage in an ecosystem where fire is expected in most years. So, overall, sympodial fragmentation offers advantages over the clonal mechanisms of typical graminoids, but it is strongly constrained by relying on external factors for its occurrence.

Interestingly, while the mechanism deviates from that of typical graminoids, it closely resembles that of Velloziaceae's arborescent monocots (Maracahipes et al., 2024). The mechanisms are so analogous that both can be termed “sympodial fragmentation,” differing primarily in some of the external factors that trigger detachment. *Vellozia* species, which range from herbaceous plants to shrubs, fragment their pseudostems when they fall to the ground due to animal activity, wind, or natural weakening (Maracahipes et al., 2024). Once on the ground, their pseudostems root themselves, much like *B. paradoxa*'s caudexes, precisely because both taxa share the distinctive internal adventitious secondary root system that absorbs moisture and grows upon contact with the soil (Mora‐Osejo, 1989; Porembski, 2006; Weber, 1963). Additionally, as clonal species, both Velloziaceae and *B. paradoxa* form large monospecific stands of aggregated populations (Maracahipes et al., 2024), enabling them to dominate spatially restricted resources that favor their growth. Coupled with quicker developmental stages and secured establishment, their clonal reproduction ensures a competitive advantage within the community (Franklin et al., 2021; Klimešová et al., 2021), reducing the risk of local extinction in these highly constraining ecosystems (Porembski, 2006; Veldman et al., 2015).

However, while clonality is the primary reproductive strategy of Velloziaceae (Maracahipes et al., 2024), it complements the sexual reproduction of *B. paradoxa*, since the species heavily invests in flowering, particularly in fire years (Fidelis et al., 2019). After a fire, ca. 65–85% of the population flowers (Miranda et al., 2024), a strategy that clearly aims to exploit the post‐fire environment and its favorable conditions for seedling recruitment (Pyke, 2017), particularly the improved thermal regimes, reduced interspecific competition, and increased nutrient availability, from which graminoids benefit (Reinke et al., 2025; Zimmermann et al., 2008). In contrast, during unburned periods, sexual reproduction is minimized to ca. 10% of individuals (Miranda et al., 2024), and environmental filters for seedlings become even harsher (Pinheiro et al., 2022), making clonal reproduction a crucial means of population maintenance. In fact, the alternative nature of clonal and sexual reproduction—dependent on varied external agents and fire occurrence—ensures population persistence even under fluctuating fire regimes or in the absence of one or more external mechanical factors. This flexible recruitment strategy highlights an adaptive advantage for persisting in an ecosystem where environmental filters significantly hinder recruitment success (Veldman et al., 2015). Thus, this dual system allows the species to capitalize on the most favorable conditions in each situation.

Our observations enhance the understanding of life strategies in the ground layer of tropical grassy biomes by contributing to a significantly understudied subject: the importance of clonal reproduction for population persistence in fire‐prone ecosystems. While previous research has established the fire‐related dynamics of sexual reproduction in *B. paradoxa*, we now show that asexual reproduction constitutes an additional mechanism for the maintenance of its populations. This dual reproductive strategy has the potential to be found in several species from these mesic grasslands and savannas, as the ground layer typically flowers in the post‐fire scenario but maintains minimal sexual reproduction during fire‐free intervals (Pilon et al., 2018, 2021). Thus, clonality can be a major trait ensuring population stability during fire‐free periods when strong competition impedes seedling recruitment.

Some questions arise from our field studies: (1) What attributes are related to an individual's clonal potential? If sympodial units are the basic unit of cloning, understanding what regulates their growth is the next step toward predicting the clonal potential of the species and its populations. (2) How long does it take for individuals to reach clonal maturity? While plants in tropical grasslands are known to be long‐lived, the time required for their life history traits to develop remains unclear. Answers to this question are essential for accurately understanding their demographics. (3) As the basic unit of cloning, can sympodial units be used as sods/plant material for restoring the ground layer? This possibility represents urgent consideration for the conservation of these grasslands, as restoring the ground layer remains one of the most challenging tasks in grassland and savanna ecology. Finally, we encourage researchers to continue investigating the persistence mechanisms and population dynamics of ground layer species and hope our study sparks renewed interest in this field.

## AUTHOR CONTRIBUTIONS

All authors conceived the ideas, designed the research, performed field work, took the pictures, and elaborated the mechanistic explanations, collectively discussing the text. HGVF created the figures and illustrations, and led the writing. All authors revised the text and gave final approval for publication.

## CONFLICT OF INTEREST STATEMENT

The authors declare no conflicts of interest.

## Supporting information


Appendix S1.
