# Supplementary material for: Beyond seeds: Revealing the clonal reproduction of Bulbostylis paradoxa as a persistence mechanism in tropical savannas
Source: Ecology. 2025 Oct 8;106(10):e70225. doi: 10.1002/ecy.70225 (PMC12505832; doi:10.1002/ecy.70225)
Supplement: Supplementary file 1 — Appendix S1. [file ECY-106-e70225-s001.pdf]

Ecology

Beyond seeds: Revealing the clonal reproduction of *Bulbostylis paradoxa* as a persistence mechanism in tropical savannas

Hudson G. V. Fontenele, Ana L. D. Lacerda, Heloisa S. Miranda

Appendix S1. Detailed description of the study sites.

Figure S1. Photographs of Sites A and B.

Site A: Área Alfa da Marinha do Brasil (AAMB), 16°00'57"S–47°55'43"W.
Photo credits: Hudson G. V. Fontenele.

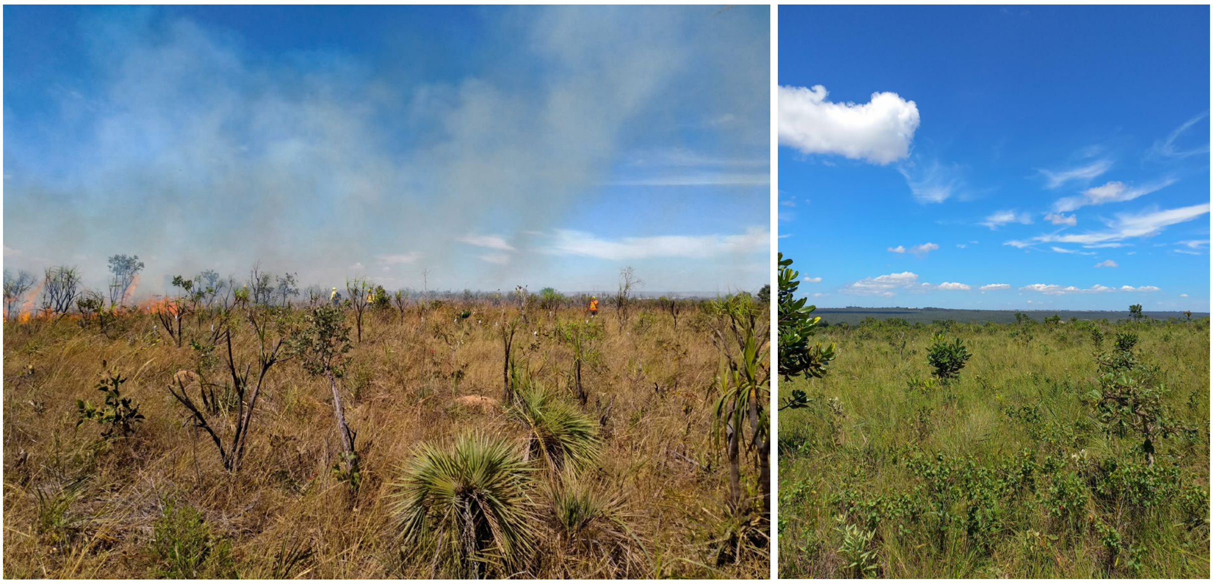

Site B: Reserva Ecológica do IBGE (RECOR IBGE), 15°57'10"S–47°52'10"W.
Photo credits: Ana L. D. Lacerda.

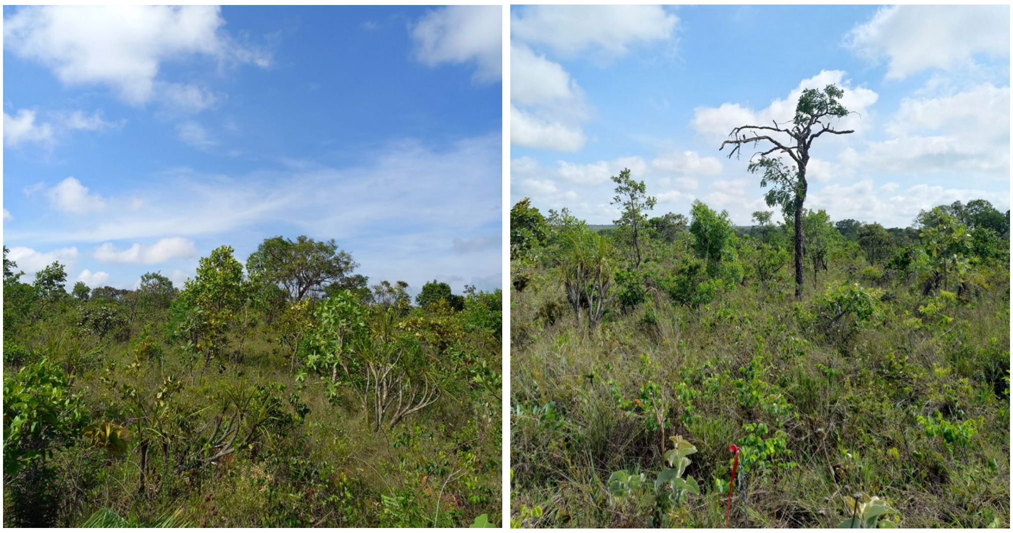

Study sites are located 10km apart within the central area of the Cerrado ecoregion in Brasília, Central Brazil. Both sites are natural old-growth grasslands (sensu Veldman et al., 2015; *campo sujo* in the local classification sensu Ribeiro & Walter). The climate is seasonal (Köppen Aw) with a marked dry season from May to September, annual precipitation of ca. 1400 mm, and mean monthly temperatures from 18 to 23°C. Soils are shallow, acidic, dystrophic, well-drained cambisols at AMMB and deep, acidic, dystrophic oxisols at RECOR IBGE.

Vegetation in both sites is characterized by a continuous ground layer dominated by C4 grasses and abundant in forbs and subshrubs. At AMMB, there is no canopy cover, and the woody layer comprises a few small shrubs and several patches of the shrub *Vellozia squamata*. At RECOR IBGE, the woody layer is slightly more prominent, but canopy cover does not exceed 15% even though bushes and shrubs are abundant.

AAMB experienced anthropogenic late-dry-season fires in 2004, 2006, 2008, 2010, 2015, and 2017. RECOR IBGE experience anthropogenic early-dry-season fires in 2002, 2004, 2006, 2008, and a late-dry-season fire in 2011. No fires were reported in both sites after the cited years.
